# Psychological Support for Family Caregivers of Patients With Amyotrophic Lateral Sclerosis at the Time of the Coronavirus Disease 2019 Pandemic: A Pilot Study Using a Telemedicine Approach

**DOI:** 10.3389/fpsyt.2022.904841

**Published:** 2022-06-16

**Authors:** Minoo Sharbafshaaer, Daniela Buonanno, Carla Passaniti, Manuela De Stefano, Sabrina Esposito, Fabrizio Canale, Giulia D’Alvano, Marcello Silvestro, Antonio Russo, Gioacchino Tedeschi, Mattia Siciliano, Francesca Trojsi

**Affiliations:** ^1^Department of Advanced Medical and Surgical Sciences, MRI Research Center SUN-FISM, Università degli Studi della Campania “Luigi Vanvitelli”, Naples, Italy; ^2^First Division of Neurology, Università degli Studi della Campania “Luigi Vanvitelli”, Naples, Italy

**Keywords:** amyotrophic lateral sclerosis, caregivers, COVID-19, telemedicine, psychological support

## Abstract

The coronavirus disease 2019 (COVID-19) pandemic confined most of the population to homes worldwide, and then, a lot of amyotrophic lateral sclerosis (ALS) centers moved to telemedicine services to continue to assist both patients with ALS and their caregivers. This pilot, randomized, controlled study aimed to explore the potential role of psychological support interventions for family caregivers of patients with ALS through resilience-oriented sessions of group therapy during the COVID-19 pandemic. In total, 12 caregivers agreed to be remotely monitored by our center since March 2020 and underwent scales for global burden (i.e., Caregiver Burden Inventory, CBI), resilience (i.e., Connor Davidson Resilience Scale, CD-RISC), and perceived stress (i.e., Perceived Stress Scale, PSS) at two-time points (i.e., at pre-treatment assessment and after 9 months or at post-treatment assessment). They were randomized into two groups: the former group underwent resilience-oriented sessions of group therapy two times a month for 3 months, while the latter one was only remotely monitored. No significant differences were found in CBI, CD-RISC, and PSS during the 9-month observation period in the treated group compared with the control group, suggesting a trend toward stability of caregiver burden together with resilience and perceived stress scores in all the subjects monitored. The lack of differences in caregivers’ burden, resilience, and perceived stress scores by comparing the two groups monitored during 9 months could be due to the co-occurrence of the COVID-19 pandemic with the stressful events related to caring for patients with ALS that might have hindered the detection of significant benefits from short-lasting psychological support.

## Introduction

Amyotrophic lateral sclerosis (ALS) is a fatal neurodegenerative and rare disease with an incidence of 1.59 per 100,000 person-years (1) that affects the motor neurons and causes progressive physical, respiratory, and swallowing impairments (2). Moreover, up to 50% of patients with ALS may develop cognitive and behavioral impairment during the disease (2) together with a high risk of severe mental disorders, which affects their function, quality of life, and mobility (3). Furthermore, the coronavirus disease 2019 (COVID-19) pandemic has affected the psychological and physical health of patients with ALS and their caregivers, leading to an increased need for assistance (4).

The patients with ALS gradually lose muscle function, thereby needing increasing care during the disease course, which mainly hurts their caregivers (5, 6). Generally, a family member (i.e., partners or sons/daughters of patients with ALS), who frequently has no previous experience in this role, may assume the role of “informal” or “family caregiver” (7). Over the past two decades, the role of family caregivers and the integration of care for patients and their families have been increasingly investigated (4, 6, 8, 9). Caregivers are crucial figures in care provision, offering emotional and physical support to the assisted patients and playing an essential role in clinical decision-making in the ALS treatment (10). On the other hand, caregivers often struggle with accepting this fatal disease, their increased responsibilities, concerns about the future, and feelings of guilt (11). Some longitudinal studies indicated that the increasing levels of motor impairment together with cognitive and behavioral deficits during the ALS progression might significantly influence caregivers’ burden (12, 13). Caregiver burden represents the impact on the emotional and physical health, the social life, and the financial status of the caregivers because of adopting the caregiving role (14). As patients are more dependent on their caregivers, this, in turn, aggravates caregivers’ negative emotions, such as anxiety. Considering the close relationship between the psychological well-being of the caregivers and the disease progression of the patients with ALS, it is crucial to monitor and treat the caregivers’ psychological status and their care burden (15). To counteract the effects of high psychological and care burdens, caregivers of patients with ALS may increase their resilience, which represents the ability to execute active/positive coping strategies in a complex scenario of different states of mind, such as those resulting from carrying out the caregiving role (16).

During the entire disease course, caregivers of patients with ALS could need to acquire management skills for supporting patients in executing cough assistance and using home ventilators, and/or promoting nutritional interventions and enteral feeding (17, 18). Therefore, patients with ALS need to have support from informal caregivers (10). However, these new duties associated with caregiving, as well as the condition of their loved ones, may have a great impact on the caregivers’ quality of life, and it reflects the importance of psychological support in the management of their condition (19). Understanding the factors associated with the caregivers can lead to more tailored support for them. The types of support could be financial, psychological, and educational relating to the condition or related to the patient’s care and supports (e.g., equipment, therapists, access to services, and respite care) (20).

As the disease’s relentless deterioration progresses, telemedicine is a valid instrument to provide care to patients with ALS and support their caregivers remotely (21). Additionally, telemedicine has become the way to deliver care and reduce the risk of more dysfunction in the current COVID-19 pandemic (22). Telemedicine aided in preserving patients’ access to clinical care and medical expertise during the COVID-19 pandemic, allowing healthcare professionals to follow-up on patients in remote locations (23, 24). Moreover, telemedicine might be a well-suited instrument for the ongoing management of such patients, particularly during a time when social distancing is encouraged (24). In particular, psychological support through telemedicine has been implemented during the COVID-19 pandemic to reduce the intensity of burden, distress, and loneliness experienced by caregivers of patients with ALS (4, 6, 22–25). Whereas extensive research has been conducted on the psychosocial aspects of caregivers of patients with ALS (2, 4, 6, 8–13, 26, 27), only a few investigations of psychological support interventions for caregivers of patients with ALS through video consults have been conducted in an ALS population, primarily in Europe, showing differences across healthcare systems, social services, and family culture (7, 22–25). In Ireland, Burke et al. (6) performed a randomized controlled trial, comparing two intervention groups, which underwent, respectively, mindfulness-based stress reduction (MBSR), used to promote the ability to cope with the management of negative emotions, and cognitive behavioral therapy (CBT), used to treat anxiety and depression, to a control group (i.e., an untreated group from a database of 75 caregivers of patients with ALS). In Italy, during the COVID-19 pandemic, the differences in social/healthcare services and approaches were more evident also in managing remotely patients with ALS and their caregivers (22–24). In Southern Italy, Capozzo et al. (22) reported the experience of a referral ALS centre by performing telephonic calls for monitoring patients with ALS, while video consults were refused due to poor practice in digital technologies for both patients and caregivers. Differently, in Northern Italy, De Marchi et al. (23) used video-calling for monitoring remotely patients with ALS and supporting their caregivers, as the approach *via* tele-consults was received as talking face-to-face to healthcare professionals. Moreover, multidisciplinary visits were provided through an online platform [IoMT Connected Care Platform (Ticuro Reply)]. Vasta et al. (24) used a mixed approach (i.e., both video and phone calls) to perform 139 neurological or psychological tele-visits, reporting substantial satisfaction with telemedicine approaches, although the majority would have preferred in-person visits.

In the present pilot study, we aimed to explore the effect of psychological support on reducing the burden and increasing the resilience of family caregivers of patients with ALS during the COVID-19 pandemic through video consults and resilience-oriented sessions of group therapy. We expected to reveal potential differences between the two groups in terms of reduction of global burden and perceived stress and an increase in resilience in the treated group compared with the untreated one.

## Materials and Methods

**FIGURE 1:**
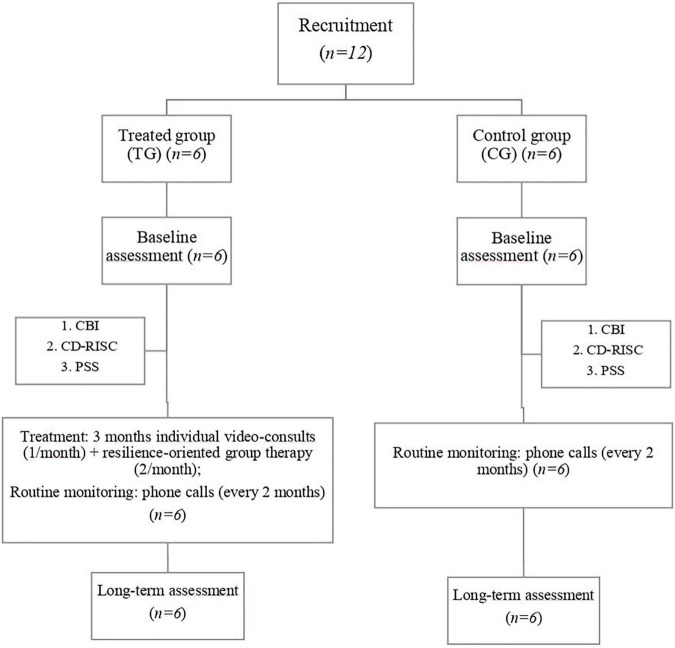
A flowchart of the recruited and monitored caregivers.

In total, 12 consecutive caregivers of patients with ALS (one each) were recruited at the First Division of Neurology of “Luigi Vanvitelli” University (Naples, Italy). The inclusion criteria were as follows: age > 18 years; being a family caregiver of a person with a diagnosis of definite or (clinically or laboratory-supported) probable ALS according to the Revised El Escorial Criteria (28); spending at least 4 h per day with the patient (10); and unimpaired cognitive performances. Caregivers with communication and hearing problems, and/or inability to comply with the study commitments were excluded. The caregiver sample was matched by the age and education level of patients with ALS. In this randomized, controlled pilot study, six consenting caregivers were randomly assigned to the treatment group (TG) and six to the control group (CG). The caregivers belonging to TG underwent regular (monthly) individual video-consults and (2 times/month) resilience-oriented sessions of group therapy in March 2020 for 3 months, immediately preceded by the administration of clinical scales at baseline. The same scales were repeated 6 months after the end of psychological support (long-term assessment). The CG group, monitored by remote phone calls every 2 months (as routinely performed in all caregivers), only completed the scales at the same time points (Figure 1). This pilot study lasted 9 months and was conducted according to the Declaration of Helsinki; informed consent was acquired from each participant by e-mail. The project was approved by the Institutional Review Board and the Ethics Committee of the University of Campania “L. Vanvitelli” (Naples, Italy).

### Description of Psychological Support

The individual tele-consults and resilience-oriented sessions of group therapy were conducted in March 2020. This treatment consisted of three individual tele-visits per participant, each lasting about 60 min, once a month for three consecutive months, combined with sessions of group therapy (2 times/month for three consecutive months), each lasting about 60 min, according to the guidelines of “counseling” formulated by the American Psychological Association (29). We adopted a model of individual, and group counseling and psychotherapy. The tele-consults/group sessions were held in a comfortable environment, by just one licensed psychologist/psychotherapist with robust expertise in ALS and cognitive disorders (DB). The primary goals were to provide non-directive support for caregivers through empathic/reflective listening and open-ended questioning. The tele-consults and group video-coaching meetings, aimed at reducing caregivers’ burden and loneliness and increasing their resilience, were focused on the physical, cognitive, and behavioral functioning and daily routines of patients; on the perceived quality of the relationship between patient and caregiver; on emotional, physical, and social burden perceived by caregivers; and on significant needs. Moreover, individual tele-consults included semi-structured qualitative interviews aimed at exploring emotions and stress perception as well as satisfaction regarding the offered telemedicine support. An interview topic guide (as shown in Supplementary Material) was used, with themes constructed through the clinical experience of research team members and literature reviews. The process for analyzing and interpreting the interviews included a thematic analysis reviewing the data for contents (in the form of transcripts, or detailed notes) (30). Thematic analysis was iterative and ongoing throughout the study (31). Interview transcripts were read in full by DB, CP, and MS and, then, were coded thematically by DB who developed a preliminary coding scheme with overarching themes and subthemes. In discussion with all researchers, a final coding framework was refined.

### Clinical Assessment

The following scales were remotely administered (in the Italian language) to all caregivers by the same licensed psychologist (CP):

• Caregiver burden inventory (CBI) (32): a 24-item multi-dimensional questionnaire measuring caregiver burden with five subscales, namely, “time dependence,” “developmental,” “physical,” “social,” and “emotional burden.” The score for each item is evaluated using a five-point Likert scale, ranging from 0 (not at all disruptive) to 4 (very disruptive), and all scores are summed; higher scores correspond to a higher burden. Cronbach’s alpha values for each subscale range between 0.73 and 0.85, and test-retest reliability is 0.94 (33).
• Connor Davidson resilience scale (CD-RISC) (34): consists of 25 statements that respondents rated on a 5-point scale from “strongly disagree” to “strongly agree.” Answers were scored from 0 to 4 to create a total score that ranged from 0 to 100, with higher numbers denoting greater resilience. Cronbach’s alpha value is 0.89, and test-retest reliability is 0.87.
• Perceived stress scale (PSS) (35): this scale assesses perceived stressful experiences or stress responses over the previous month with a 5-point Likert scale (0 = never and 4 = very often). PSS-10 scores are obtained by reversing the responses (e.g., 0 = 4, 1 = 3, 2 = 2, 3 = 1, and 4 = 0) to the four positively stated items (items 4, 5, 7, and 8) and then summing across all scale items. The scores range from 0 to 40, with higher scores indicating greater stress. Cronbach’s alpha value is 0.74, and test-retest reliability is 0.85 (36).

Patients with ALS were assessed by a disability score (i.e., ALS Functional Rating Scale-revised, ALSFRS-R total score, where lower total reflects higher disability) (37) and a global cognitive functioning score (i.e., Edinburgh Cognitive and Behavioral ALS Screen, ECAS) (38).

### Statistical Analysis

At the pre-treatment assessment, the study groups on demographics and clinical measures of the cared-for patients were compared *via* independent *t*-test or Pearson’s chi-squared test (χ^2^ test) when appropriate. Moreover, the pre-treatment differences in CD-RISC, CBI, and PSS were explored *via* an independent *t*-test. In line with O’Connell et al. (39), to test the effects of psychological support on CD-RISC, CBI, and PSS, the two study groups were compared on the post-treatment-assessment measures by analysis of covariance (ANCOVA), using the pre-treatment measures as covariates. When we carried out multiple comparisons between treated and untreated study groups, the Bonferroni correction to the alpha level was applied to avoid Type I error. All analyses were performed using the IBM Statistical Package for Social Science (SPSS) version 20.

## Results

**FIGURE 2:**
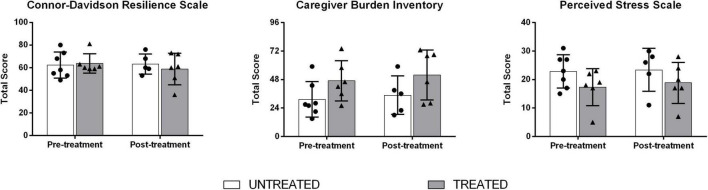
Between-group comparison on Connor-Davidson Resilience Scale (CD-RISC), Caregiver Burden Inventory (CBI), and Perceived Stress Scale (PSS) scores at pre- and post-treatment.

At the pre-treatment assessment, the two groups of patients with ALS associated with the two studied groups did not differ in demographics or clinical measures (i.e., ALSFRS-R, *F*-statistic = 0.60, *p*-value = 0.556 and ECAS scores, *F-statistic* = –0.08, *p*-value = 0.937; Table 1). Moreover, no statistically significant differences were found in pre-treatment measures of the CD-RISC (*F*-statistic = –0.27, *p*-value = 0.792), CBI (*F*-statistic = –1.77, *p*-value = 0.103), and PSS (*F*-statistic = 1.61, *p*-value = 0.134; Table 2 and Figure 2).

**TABLE 1:** Between-group comparison at pre-treatment assessment; data are reported as mean ± standard deviation (SD) or count (percentage).

Variable	Untreated (CG)	Treated (TG)	χ^2^ test^a^; *t*-test^b^	*p*-value^c^
Caregivers’ sex (male)	3 (42.9%)	2 (33.3%)	0.12^a^	0.797
Caregivers’ age at interview, years	53.29 ± 10.48	60.33 ± 8.98	−1.28^b^	0.224
Caregivers’ years of education	12.00 ± 2.94	11.83 ± 3.18	0.09^b^	0.924
**Relationship with patient:**			0.25^a^	0.612
Husband/wife	5 (71.4%)	5 (83.3%)		
Son/daughter	2 (28.6%)	1 (16.7%)		
Patients’ sex (male)	3 (42.9%)	3 (50.0%)	0.06^a^	0.797
Patients’ age at assessment, years	57.50 ± 6.02	60.00 ± 8.94	−0.56^b^	0.583
Patients’ years of education	8.60 ± 4.56	8.50 ± 2.58	0.04^b^	0.964
Age at onset, years	54.17 ± 11.75	56.67 ± 10.03	−0.39^b^	0.700
Duration of disease, months	52.50 ± 75.49	41.83 ± 23.08	0.33^b^	0.747
ALSFRS-R	24.71 ± 13.53	21.00 ± 6.81	0.60^b^	0.556^a^
ECAS-CS	92.67 ± 18.82	93.60 ± 13.50	−0.08^b^	0.937

*ALSFRS-R, ALS Functional Rating Scale-Revised; CG, control group; ECAS-CS, Edinburgh Cognitive and Behavioral ALS Screen-Cognitive Score; TG, Treated Group.*

*^a^Marks the results of the “χ^2^ test” and ^b^of the “t-test”. ^c^Bonferroni corrected alpha level of 0.05/11 = 0.005.*

**TABLE 2:** Between-group comparison on psychological measures at pre- and post-treatment assessment (using pre-treatment assessment as covariate).

Variable	Untreated (CG)	Treated (TG)	*t*-test^a^; ANCOVA^b^	*p*-value*^c^*
**Connor-Davidson Resilience Scale**				
Pre-treatment	62.2 ± 11.51	63.83 ± 8.54	−0.27^a^	0.792
Post-treatment	63.30 ± 8.92	58.83 ± 13.87	0.25^b^	0.629
**Caregiver Burden Inventory**				
Pre-treatment	31.29 ± 14.90	47.00 ± 16.95	−1.77^a^	0.103
Post-treatment	34.80 ± 16.20	51.83 ± 21.08	0.29^b^	0.601
**Perceived Stress Scale**				
Pre-treatment	22.86 ± 5.84	17.33 ± 6.47	1.61^a^	0.134
Post-treatment	23.40 ± 7.53	18.83 ± 7.19	0.02^b^	0.888

*t-test was used for comparing the Untreated and Treated groups on pre-treatment measures; Analysis of Covariance (ANCOVA) was used for comparing the Untreated (CG) and Treated groups (TG) on post-treatment measures, using the pre-treatment ones as covariates.*

*^a^Marks the results of the “χ^2^ test” and ^b^of the t-test. ^c^Bonferroni corrected alpha level of 0.05/6 = 0.008.*

At the post-treatment assessment, CG and TG did not differ on CD-RISC (*F*-statistic = 0.25, *p*-value = 0.629), CBI (*F*-statistic = 0.29, *p*-value = 0.601), and PSS (*F*-statistic = 0.02, *p*-value = 0.888; Table 2 and Figure 2). The treated caregivers represented 83% of spouses (four women; mean age 60.3 + 8.9 years), who accepted to share their most intimate, sensitive, and vulnerable parts. Through our data analysis, three overarching themes were generated: (1) activities of a caregiver of a patient with ALS; (2) changing dynamics of care and connectedness among family and friends; and (3) satisfaction regarding the health services offered by our center. Regarding themes 1–2, semi-structured qualitative interviews, performed during individual tele-visits, revealed that caregivers dealt with uncertainty, unpredictability, helplessness, and frustration and found themselves lacking even those few, but indispensable, social resources that make the difference in everyday life. Regarding theme 3, all the included caregivers declared that they were satisfied with the services of our center during the COVID-19 pandemic, although they would have preferred integration with in-person visits.

## Discussion

This pilot study aimed at exploring if psychological support by telemedicine services for ALS caregivers during the COVID-19 pandemic was useful to reduce caregivers’ burden and their perceived stress by improving caregivers’ resilience to cope with the disease condition. However, our study showed that short-lasting psychological support by telemedicine during a pandemic was not enough to help more ALS caregivers in that no significant differences were found between TG and CG regarding changes in global burden, resilience, and perceived stress scores across time. To note, caregivers’ burden and perceived stress scores did not increase across 9 months in either monitored group, as well as resilience measures.

In agreement with our findings, de Wit and colleagues revealed that tailored support programs for caregivers of patients with ALS and progressive muscular atrophy (PMA) (i.e., a blended intervention through face-to-face contact and e-health, based on Acceptance and Commitment Therapy or ACT) did not reduce their distress, but may be beneficial by increasing the feeling of control in caregiving situations (40). The participants positively evaluated this protocol: caregivers referred that it helped them to be more aware of their situation and to perceive more control over it, empowering caregivers to make choices according to their own needs. The online approach was also appreciated: caregivers may experience a lack of personal time, since they spend many hours providing care, especially in the advanced stages of ALS. Using the online support enabled them to enter the program at their preferred time and place. Additionally, Tang et al. (41), carrying out a face-to-face interview in 120 pairs of patients with ALS and their caregivers, revealed that higher anxiety index scores were associated with greater caregiver burden, as well as previously demonstrated also regarding the association between depression and caregiving burden in ALS (42). These findings altogether suggest that the level of disease knowledge, anxiety, and depression may be associated with caregiver burden, indicating the need for support programs to alleviate this burden.

Regarding the impact on caregivers’ anxiety/depression and burden of the COVID-19 pandemic, our findings did not reveal a significant increase in perceived stress and burden in either monitored groups, despite the imposition of national quarantine and other social restrictions that have induced most caregivers to perceive more loneliness (8) and a worsening of homecare assistance (19). Although the 3-month psychological support protocol reserved for TG did not show significant benefits in caregiver burden and distress, the remote bi-monthly phone calls that we targeted to all patients and caregivers (including those from the studied TG and CG) may have reduced perceived loneliness and subsequent distress. Possible interpretations of this negative result could be related to the small sample size, the relatively short time of the psychological support, and the intrinsic difficulties of implementing effective psychological support for patients with ALS and their caregivers due to the rapid and critical progression of the disease and the severe care needs. In fact, in our sample, three patients assisted by caregivers belonging to TG died due to disease progression during the 3-month treatment, as did one patient assisted by a caregiver belonging to the CG, although the two studied groups were matched for patients’ disease duration, disability (i.e., ALSFRS-R score) and cognitive performance (i.e., ECAS score) (Table 1). Therefore, the caregivers of patients with ALS should be informed about possible supportive interventions at an early stage of the disease and offer these interventions repeatedly (40). Moreover, offering customized care in line with the caregiver’s preferences would be advised (40). As for our monitoring of resilience measures in the studied groups, the lack of changes in CD-RISC scores across time in both TG and CG may be due to the recognized co-existence and interrelation of burden, resilience, needs, and rewards in caregivers of patients with ALS (16). In this regard, Weisser et al. (16) identified a model of coping for caregivers of patients with ALS that integrates resilience (active/positive), burden (active/negative), needs (passive/negative), and reward (passive/positive) to develop appropriate individualized caregiver support plans for increasing resilience.

## Limitations and Future Directions

Our pilot study has several limitations: data were collected from a clinic hospital and a small sample of caregivers; thus, our findings may have limited generalizability; the time and number of interventions were too limited; the small sample of caregivers was primarily represented by women; and scales for depression and anxiety were not monitored, primarily aiming to reveal potential changes in resilience and perceived stress across time, in accordance with the tailored, psychological intervention performed. However, most limitations of our study have been frequently shown in most literature concerning psychological interventions for patients with ALS (43). A recent scoping review (43) revealed that the existing studies addressing this topic, which included three randomized-controlled trials and some observational studies, focused on a limited number of psychological outcomes, thus requiring further evaluation. Therefore, future studies are needed to examine the associations between patients’ outcomes and family caregivers’ psychological support needs and information-seeking behaviors. In addition, the preferences regarding these psychological support sources, such as telemedicine, as well as the accuracy of each source, should be evaluated. It will be necessary to evaluate the effects of combined approaches, such as in-person care and remote psychological support, on the well-being of caregivers of patients with ALS and to implement the number and frequency of the group-therapy sessions and the televisits.

## Conclusion

The co-occurrence of the COVID-19 pandemic and stressful events related to caring for patients with ALS may influence the response to psychological interventions aimed at reducing caregivers’ burden and perceived stress and at increasing resilience. Moreover, a combination of both remote and in-person approaches would be needed in emergencies as well as in routine conditions. In particular, the COVID-19 outbreak, which prompted the more widespread use of telemedicine services, has allowed experience that telemedicine should be intended to be complementary to in-person care in managing patients with ALS.

## Data Availability Statement

The raw data supporting the conclusions of this article will be made available by the authors, without undue reservation.

## Ethics Statement

The studies involving human participants were reviewed and approved by the Institutional Review Board and the Ethics Committee of the University of Campania “L. Vanvitelli” (Naples, Italy). The patients/participants provided their written informed consent to participate in this study.

## Author Contributions

FT, MSic, and DB: conceptualization. FT, MSic, DB, CP, MD, GD’A, FC, SE, MSil, and AR: methodology. CP, MD, GD’A, FC, SE, MSil, and AR: investigation and data collection. MSh and FT: writing—original draft preparation. MSh, FT, MSic, DB, and GT: writing—review and editing. FT, MSic, and GT: supervision. All authors have read and agreed to the published version of the manuscript.

## Conflict of Interest

The authors declare that the research was conducted in the absence of any commercial or financial relationships that could be construed as a potential conflict of interest.

## Publisher’s Note

All claims expressed in this article are solely those of the authors and do not necessarily represent those of their affiliated organizations, or those of the publisher, the editors and the reviewers. Any product that may be evaluated in this article, or claim that may be made by its manufacturer, is not guaranteed or endorsed by the publisher.
