# Supplementary material for: Psychological Support for Family Caregivers of Patients With Amyotrophic Lateral Sclerosis at the Time of the Coronavirus Disease 2019 Pandemic: A Pilot Study Using a Telemedicine Approach
Source: Front Psychiatry. 2022 Jun 16;13:904841. doi: 10.3389/fpsyt.2022.904841 (PMC9243390; doi:10.3389/fpsyt.2022.904841)
Supplement: Supplementary file 1 [file Data_Sheet_1.PDF]

<u>TOPIC GUIDE: INTERVIEW WITH CAREGIVERS OF ALS PATIENTS DURING COVID-19 PANDEMIC</u>

<u>Caregiver profile</u>

- Age, gender
- Past occupation/present occupation and hobbies
- Living situation

<u>Daily routines</u>

- Being a caregiver of a patient with ALS, what did a typical day look like before COVID-19?
- What does a typical day during the COVID-19 pandemic look like? What has changed?

<u>Social network</u>

- Are you part of a community/association/social group?
- With whom did/do you regularly keep in contact?
- What ways of contact do you have? What different communication methods do you use now with your loved ones?
- Many patients and caregivers express a sense of isolation during lockdown. What is your view on that, also considering your condition of caregiver of a patient with ALS?
- When did you decide to self-isolate? How has this self-isolation and physical distancing affected your social networks (i.e. community groups, friends, family)
- Do you regularly get phone calls from family/friends? Has this always been the case, or has it changed since COVID-19?

<u>Vulnerability</u>

- What do you think of the guidelines calling for severely disabling patients to self-isolate?
- Do you feel “vulnerable”?
- What are your views about the media reporting around COVID-19? What about the portrayal of risk attributed to severely disabling people?

<u>Topics important to the participant and satisfaction regarding the telemedicine services offered</u>

- Which topics have we not covered that are important around COVID-19?
- What do you think about our tele-consults/group sessions and individual tele-visits?
- What do you think about our phone calls to monitor remotely ALS patients during lockdown?
- Would you prefer the combination of telemedicine and in-person visits?

Is there anything else you would like to add?
